# Grape Pomace Valorization: A Systematic Review and Meta-Analysis

**DOI:** 10.3390/foods9111627

**Published:** 2020-11-07

**Authors:** Bojan Antonić, Simona Jančíková, Dani Dordević, Bohuslava Tremlová

**Affiliations:** 1 Department of Plant Origin Foodstuffs Hygiene and Technology, Faculty of Veterinary Hygiene and Ecology, University of Veterinary and Pharmaceutical Sciences, 61242 Brno, Czech Republic; antonicb@vfu.cz (B.A.); jancikovas@vfu.cz (S.J.); tremlovab@vfu.cz (B.T.); 2 Department of Technology and Organization of Public Catering, South Ural State University, Lenin Prospect 76, 454080 Chelyabinsk, Russia

**Keywords:** byproduct, waste management, food fortification, total dietary fiber, polyphenolic content, grape pomace

## Abstract

This systematic review aimed to collect data and analyze the possible use of grape pomace, a winemaking industry byproduct, in the production of fortified foods. The English articles found in Web of Science, Scopus, and Google Scholar, from January 2006 until May 2020, were used for the conduction of overview tables and meta-analysis. The systematic review emphasized the two main issues concerning grape pomace application to other food products: (i) grape pomace contains high amounts of health promoting compounds; and (ii) the use of grape pomace is influencing the waste management. The grape pomace has been used in the fortification of plant origin food, meat, fish, and dairy products, mainly due to higher polyphenols and dietary fiber contents. The fortification was declared as successful in all studied food types. The change of color, caused by polyphenolic compounds, was mainly observed as an adverse effect of the fortification. Higher levels of fortification also caused notable undesirable changes in texture. The most valuable influence of the grape pomace addition according to included papers and meta-analysis is certainly a higher nutritional quality and oxidative stability of fortified products, reflected as higher polyphenol and total dietary fiber content.

## 1. Introduction

Grapes are one of the most produced crops worldwide with the production estimation of more than 79 million tons in 2018, according to the Food and Agriculture Organization (FAO—United Nations) [1]. Grape consumption was found to be beneficial for human health due to the large content of bioactive substances [2]. Approximately 75% of produced grapes is intended for wine production, out of which 20–30% represents waste products [3,4]. This waste is also called grape pomace and consists of skins, remaining pulp, seeds, and stalks [5]. These byproducts represent waste disposal or they are used for wine alcohol production, serve as fertilizer or as animal feed [3]. The disposal of this waste creates environmental problems such as pollution of ground and surface water, the attraction of disease-spreading vectors, and oxygen consumption in soil and groundwater which can have an impact on wildlife [6]. Large quantities of grape pomace disposed of in landfills during the harvest season can have negative effects on biodegradation due to low pH and the presence of antibacterial substances, such as polyphenols [7]. Though, grape pomace is rich in proteins, it was reported that most of the animals cannot digest it and use it as a source of energy [8]. The use of the grape pomace as a composting material is not economically viable due to a lack of some essential nutrients [6]. On the other hand, grape pomace contains significant amounts of substances that can be considered beneficial to health [9,10]. The most abundant in grape pomace are dietary fibers that are present in high levels (up to 85% depending upon the grape variety) and polyphenolic compounds that mainly (about 70%) remain in pomace after the winemaking process [11,12].

Grape pomace waste management represents an important environmental issue. On the other hand, there is an increasing demand for healthy and natural food ingredients that can replace synthetic antioxidants and food preservation substances [13]. The fact that grape pomace possesses the biotechnological potential, created many studies that have been dealing with the possibility of using it as the fortification ingredient in food [14]. In these studies, grape pomace has improved the nutritional profile of the final product and increased its value. The wide range of products was covered in those studies, including plant food products, meat products, fish, and dairy products. Grape pomace was used in production from only 0.06% (pork burgers, Garrido et al. [13]) to 100% (tea infusion, Bekhit et al. [15]).

This systematic review aimed to investigate the results of studies that included grape pomace in the production of functional food. Fortification levels were identified, and their impact on the final product quality, the same as the problems (change in color, texture, and taste) that occur in the fortification process. As a quantitative synthesis of data from various studies, meta-analysis gave a clearer picture about the use of grape pomace as a functional part of different food products. According to the present review papers (which can be found in relevant databases: Web of Science and Scopus), a systematic review analyzing via meta-analysis the inclusion of grape pomace as the fortification element in different edible matrices has never been done before. Possibilities and limitations were clearly elaborated, giving a contribution to future studies.

## 2. Search Strategy and Methodology

The scientific literature published in the English language was used in the systematic review. Sources that were used to collect the target articles were Web of Science, Scopus, and Google Scholar. Keywords used in Web of Science and Scopus were “grape AND pomace”, obtained search results were preselected manually based on the names of the found articles. Google Scholar was searched only by titles using the search term “allintitle: grape AND [pomace OR byproduct OR by-product OR fortification OR fibers OR fiber]”. A total of 1703 articles from Web of Science, 1276 articles from Scopus, and 836 from Google Scholar were obtained in the search. The manual selection was sufficient to obtain enough data for making the proximate composition table, tables describing the effects of fortification of different food commodities with the grape pomace, and for the conducting the meta-analysis. Articles used in this review were from January 2006 to May 2020. A total of 18 articles were used for the creation of a proximate composition table, and 38 studies were used for a table about the use of grape pomace as a fortification ingredient in different kinds of products.

Meta-analysis was done using the Review Manager Software (version 5.3, developed by the Cochrane Collaboration, London, UK).

## 3. Proximate Composition of Grape Pomace

The composition of the grape pomace as a waste product is highly dependent on the type of waste, grape variety, planting environment, processing method, and many other factors [13]. While the red wine production includes fermentation of the whole grape mass, the rose and white wines are made by juice fermentation. All this leads to the high variation in grape pomace composition and represents a significant challenge for the grape pomace valorization and fortification processing [16,17].

The proximate composition of grape pomace obtained from studies that included an analysis of different varieties of red and white grape pomaces is presented in Table 1.

The most important constituents of grape pomace are fibers, polyphenolic compounds, colorants, and minerals. Polyphenolic compounds, colorants, and anthocyanins are the main carriers of the grape pomace antioxidant potential. The oily part of the grape pomace is rich in unsaturated fatty acids, colorants, and minerals. Immediately after production, grape pomace contains large quantities of water, which affects its chemical stability and favors microbial spoilage. Consequently, it is very important to dry grape pomace and slow down those processes [3].

Dietary fibers were found as predominant compounds in red grape pomaces, though in white grape pomaces dietary fiber content is significantly reduced. Soluble sugars are the largest constituent in white grape pomace [25]. The main part of dietary fibers comprises insoluble fibers like cellulose and hemi-celluloses. Insoluble fibers are characterized by high porosity and low density, improving the efficiency of the digestive tract [4]. Some fiber compounds in grape pomace make chemical bonds with phenolic substances and, thus, create antioxidant dietary fibers, giving the pomace stronger radical scavenging potential. This gives them a higher nutritive value in comparison to dietary fiber present in cereals. Studies confirmed the greater effect of these complex compounds with dietary fibers on human health [23]. Since fibers from grape skin consist of lignin, cellulose, hemicellulose, these compounds represent the source of supporting materials [12].

During grape processing, present polyphenols mainly remain in the grape pomace due to their incomplete extraction. The main representatives of polyphenolic compounds in this byproduct are anthocyanins (only in red grape pomaces), catechins, flavonol glycosides, phenolic acids, and alcohols [31]. Together with dietary fibers, phenolic compounds are the most valuable compounds of grape pomace with health beneficial properties, such as the maintenance of intestinal health and the prevention of chronic diseases and cancer [9,32]. Many studies showed the great antioxidant potential of polyphenols and their use in food preservation due to the inhibition of lipid oxidation and antibacterial effect [33]. The mechanisms of antioxidant activity are based on their structure and include the radical scavenging ability, electron donation, or chelation of metal ions [22]. Anthocyanins are red color pigments susceptible to changes due to the light, temperature, pH, or some other external factors [34]. The stabilization of these pigments would represent a valuable source of natural colorants in the food industry [12]. Flavanols are the main bioactive compounds in white pomace since anthocyanins are absent. Up to 65% of the total grape flavanols content was found in the seeds; in skins, the total flavanol content increases to 21% [3].

The content of the soluble sugars glucose and fructose in red grape pomaces is usually low because of the red wine production process and these sugars are mainly consumed by yeasts in the fermentation process. Higher glucose and fructose contents (up to 26.34 and 8.91 g/100 g, respectively) are found in white grape pomaces, because of which it can be used to enhance the sweet taste in fortified products [17,35].

The grape seeds compose of 40% fibers, 10–20% lipids, 10% proteins, and the rest are sugars, polyphenolic compounds, and minerals [12]. The most important constituent of grape seed is oil, rich in unsaturated fatty acids with linoleic and oleic acid [36]. Additionally, there is a significant amount of vitamin E, sterols, and other bioactive compounds that possess antioxidant and anti-cancerogenic activity [37,38].

Regarding the content of essential mineral, iron and zinc were found in wide ranges: 5 mg/100 g to 5468 mg/100 g and 2 mg/100 g to 2254 mg/100 g, respectively. These two minerals are found to have a large impact on the antioxidant potential, too [2]. Grape pomace can be considered as a good source of potassium since its levels ranged up to 3157 mg/100 g. Potassium plays an important role in lowering blood pressure and decreasing the risk of osteoporosis due to the reduced urinary calcium excretion [2]. 

In addition to health-beneficial compounds, grape pomace might contain health-hazardous compounds as well. Those are mycotoxins, including ochratoxin A, which is classified as carcinogenic. Ochratoxin A on grape crops is mainly produced by *Aspergillus carbonarius* [39,40]. The production of this mycotoxin is influenced by climatic conditions, grape variety, crops damage, and other factors. More than 90% of ochratoxin A in grape processing retains in grape pomace. Consequently, if grape pomace is used as the ingredient in certain food commodities, it can represent a health hazard due to the possible presence of ochratoxin A. Its thermal stability even at temperatures up to 250 °C makes it difficult for elimination in heat-processed food [41]. The levels of the ochratoxin A, in analyzed grape pomace samples, were approximately 0.07 μg/kg. This amount is not considered a threat to human health, since it is lower than 2 μg/kg, the limit given by the European Union [40,42].

## 4. Studies about the Use of Grape Pomace as a Fortifying Agent

### 4.1. The Fortification of Plant Foodstuffs

Those products included muffins, cookies, biscuits, bread, extruded cereals, noodles, pancakes, pasta, and tomato puree. The summary of those studies is presented in Table 2.

Several studies included muffins as the fortified product [4,43,44]. In the work of Bender et al. [4], tests on muffins were run by the replacement of wheat flour with 5, 7.5, and 10% of grape skin flour, obtained from red (Tannat) and white variety (Riesling). Dietary fibers were successfully transferred to the final product with a statistically significant increase in all used percentages. The final fortified products were darker; red varieties had higher impact due to the initial flour darker color. However, the textural analysis revealed an increase of hardness and cohesiveness; sensory analysis showed no significant change in consumers’ perception in all tested samples. The muffin fortification conducted by Ortega-Heras et al. [43] was done by the replacement of wheat flour with 10 and 20% of grape pomace, obtained from red and white grape variety. The effect of fortification was similar to the study conducted by Bender et al. [4], since the increase of dietary fiber and changes in color and texture were observed. The addition of 20% of grape pomace significantly decreased liking among consumers. In the study of Walker et al. [44], the increase of total phenolic content in fortified muffins was observed.

Fortification of bread was also an interesting topic in the studies [21,44,45,46,47,48]. In those studies, flour was replaced from 2% in the work of Hayta et al. [46] to the highest 15% in the work of Walker et al. [44]. In all mentioned studies a value-added bread with increased total phenolic content and radical scavenging activity was successfully made. The incorporation of both red and white grape pomaces resulted breads with darker crust and crumb. Tannins and anthocyanins are the main polyphenolic compounds responsible for the red, purple, and blue color in red grape cultivars. In white wines, hydroxycinnamic acids represent compounds that contribute to oxidative browning, and flavonols influence the occurrence of the yellowish color [56,57]. Thus, bread with added red grape pomace had a darker reddish color, while bread with added white grape pomace had brown to yellow notes of color, depending on the fortification level. There was a noticeable decrease in the loaf volume of fortified products. This could be explained by the increased content of dietary fiber and consequently increased water absorption [44,48]. Additionally, the increase of polyphenols might affect the enzymes and yeasts in the dough formation process [35,44].

Theagarajan et al. [20] researched the shelf life of the cookies fortified with 4 and 6% of grape pomace. During the 60 days of the storage period, the hardness of cookies, the property associated with product freshness, was higher in fortified samples. Moreover, in cookies containing grape pomace, the lipid oxidation was inhibited, too. This may be the result of an increased antioxidant activity that prevents peroxidation [20].

Tomato puree was also studied while being fortified with grape skin powder in a concentration of 3.2%, using the different particle sizes [54]. Tomato contains lycopene, which is a strong antioxidant, but the study proved that the antioxidant activity of tomato puree samples prepared with the addition of grape pomace had a higher antioxidant potential in comparison with samples without grape pomace. Additionally, heat treatment did not have a negative effect on polyphenol concentrations. The sensory analysis confirmed that the lower particle size of grape skin powder gained better results among the panelists due to better textural properties [54].

Studies about using the grape pomace to enrich pasta were also conducted. Marinelli et al. [52], used grape pomace water extract to replace the water in the pasta making process. The results revealed an increase in total phenolic content and antioxidant activity, and there were no significant differences in sensory properties. A different approach was taken by Sant’Anna et al. [53], where grape pomace powder was added in concentrations of 2.5, 5, and 7.5% to the pasta recipe. The increase of total polyphenolic content and antioxidant activity in fortified products was observed. The addition of grape pomace influenced the color, but the sensory analysis revealed that the addition of 2.5% had very similar marks to the control sample and they were equally accepted among panelists. In the study of Gaita et al. [55], besides an increase in the antioxidant activity, there was also an increase in sensory properties of samples fortified up to 6% with grape skin flour.

The study conducted by Bekhit et al. [15], included the tea infusions preparations made from grape skins of pinot noir (red grape variety) and pinot gris (white grape variety). Though, the mentioned extracts had weaker antioxidant activity in comparison to hibiscus and green tea, they exhibited strong antiviral activity. The antiviral activity was not related to the phenolic content [15].

The technologies applied for making fortified (grape pomace as the fortification element) plant origin products included heat treatment up to 220 °C, during 10 min, for bread making process [46] or at temperatures less than 200 °C for the time period below 20 min [21,44]. The obtained results indicated that these conditions (heat treatments) did not have a negative impact on final fortified products. Studies also confirmed the increase of total polyphenolic content in samples that were heat-treated. The increase of polyphenolic compounds can be explained with the fact that many polyphenols are linked to other compounds. At higher temperature, polyphenolic compounds might be released and become more available [58,59]. It should be mentioned that the particle size of the added ingredient plays an important role as well: (a) extractability of bioactive compounds, such as polyphenols, is increased; and (b) higher consumers’ acceptance since textural parameters are influenced positively by smaller particles [54].

### 4.2. The Fortification of Meat and Fish Products

The grape pomace was used for the fortification of meat and fish products. Studies included pork burgers, beef frankfurters, pork sausages, pork loin marinade, chicken meat, salmon burgers, and minced fish muscles. The summary of the above-mentioned studies is presented in Table 3.

The studies involving the fortification of meat and fish products mainly aimed to investigate the effect of grape pomace inclusion on products’ shelf life, storage stability and lipid oxidation. The fortification was done by the addition of grape pomace powder into the recipe [19,62,67], the addition of grape pomace extract to the product [13,61,63,64,65] or by soaking the product in the marinade containing the grape pomace solution [60,66].

In the study by Ryu et al. [62], cooked pork sausages were enriched with 0.5 and 1% of grape pomace. The significant decrease in color lightness was observed in both fortified formulations with a higher influence in samples containing higher levels of grape pomace. The redness was increased that can be attributed to the anthocyanins present in the grape skins. TBARS (thiobarbituric acid reactive substances) was employed in samples to check lipid oxidation during storage time; the samples containing 0.5% of grape pomace had the lowest TBARS values after 10 days of refrigerated storage [62].

Garrido et al. [13], enriched pork burgers by adding to the recipe 0.06% of grape pomace extracts. Microbial tests showed no significant differences between control and fortified samples, probably because of low extracts concentration. On the other hand, lipid oxidation results showed significantly lower values for fortified samples. Even in low concentrations, extracts containing higher amounts of anthocyanins affected the color of the product and exhibited higher color stability in comparison with control samples [13].

Seed extract in six concentrations was used for the enrichment of beef frankfurters in the work of Özvural and Vural [61]. The addition of extract into frankfurters recipe suppressed the lipid oxidation in comparison to the control sample. There was a significant difference in color between all the samples, and two formulations with the highest concentrations were found less acceptable than all other ones among panelists [61].

The study of Lee et al. [60], investigated the impact of grape pomace marinade on the pork loin meat quality. In their experiment, 100 g of raw meat was marinated in 1 L of water solution, containing grape pomace in concentrations of 0.5, 1, 2, 20, and 40%. The color change was observed, and all samples were lighter than control samples. Regarding other color parameters, the highest concentration of grape pomace marinade affected meat color to be more reddish and yellowish than control samples. After 10 days of storage, it was concluded that grape pomace marinade inhibited lipid oxidation in the meat product [60].

Fortification of fish products was studied by Cilli et al. [19] where 1% and 2% of grape pomace were added to the salmon burger mixture. The addition of grape pomace influenced the increase of dietary fibers in burgers. Regarding the color, as in meat products, a darker color of the final product was observed in enriched products. TBARS values clearly showed that added grape pomace in both concentrations had a protective role against lipid oxidation. Sensory analysis revealed lowered consumers’ acceptance in comparison to the control. Similar observations regarding the preservation role of grape pomace on fish products were conducted in studies of Sanches-Alonso et al. [67] and Ozalp et al. [64].

The chicken meat was effectively fortified in studies involving soaking in a marinade containing a grape seed extract at a concentration of 2500 ppm [66] or the addition of grape pomace extract to the minced chicken meat products in order to achieve a TPC of up to 60 mg/kg [63,65]. All mentioned studies revealed the decrease in lipid oxidation due to the strong antioxidant activity; the soaking in the grape pomace had also a positive effect on the chicken meat texture. Sensory evaluation from the study conducted by Selani et al. [63], revealed changes in color and characteristic chicken meat odor.

### 4.3. The Fortification of Dairy Products

Yogurt was the main product among dairy products used in studies including fortification. Table 4 summarizes the impact of grape pomace on yogurt, cheese, fermented milk, and ice cream.

The studies that included yogurt as a fortified product used grape pomace flour [22,72], grape pomace extract [22,70], grape skin flour [74], and grape seed extract [69,71] for the fortification.

In the work of Tseng et al. [22], grape pomace was added to the coagulated milk in concentrations of 1, 2, and 3%. The total dietary fibers content was increasing with higher concentrations of grape pomace. Fortified yogurts exhibited color with lower lightness, increased redness, and blueness. These properties were expected due to the dark color of red grape pomace. The decrease in viscosity was observed in treated yogurts, while syneresis was stable during the four weeks of storage. Inhibition of lipid oxidation was also reported [22].

Grape pomace powder was used in the study of Demirkol et al. [72], where it was added in concentrations of 1, 3, and 5% to the milk with yogurt cultures before the fermentation. The fortification was successful in the sense of the total phenolic content and radical scavenging activity increment. On the other hand, the color was significantly affected, same as the viscosity of the samples. The decrease of viscosity with the increase of fortification degree was observed, and this might be due to the weakening of the yogurt gel by higher concentrations of the grape pomace. Sensory analysis revealed higher consumers’ acceptance of control samples prepared without grape pomace addition [72].

The addition of grape pomace extract (1%) originated from different grape varieties in the work of Karaaslan et al. [70] increased antioxidant capacity and total polyphenolic content of treated yogurt. There was a slight color change, but sensory characteristics were not affected significantly. The works including the use of grape seed extract to the milk before the fermentation, positively affecting the total polyphenol content, with the expected impact on the final product color [70].

The highest degree of yogurt fortification was done in the work of Marchiani et al. [74], where 6% of grape skin flour was incorporated in the yogurt formulation. Though the total phenolic content and antioxidant activity were increased, notable undesirable changes were observed in the texture and sensory perception among panelists due to the sour taste [74].

The fortification of semi-hard (Toma-like) and hard (cheddar) cheeses with 0.8 and 1.6% of grape pomace resulted in increased antioxidant activity and total phenolic content. No changes were reported regarding physicochemical properties and microbial counts, marking the fortification as successful [74].

The study of Frumento et al. [68] included fermented milk fortification and showed that different grape pomaces have significantly different effects on the milk fermentation and final product physicochemical properties. The selected grape pomace flour was added at concentration of 2% to the milk before fermentation. The fortified fermented products exhibited higher phenolic content and antioxidant activity. Additionally, a shorter fermentation period was observed in samples containing the grape pomace, which can be attributed to the increased sugar content and lower initial pH [68].

Grape juice residue was also used as a fortification ingredient and can be named grape pomace. Vital et al. [34] were preparing the ice cream with the incorporation of this byproduct in the following concentrations: 2.5%, 5%, and 10%. Even though polyphenolic compounds are more susceptible to degradation at freezing temperatures, all fortified samples showed a significant increase in antioxidant activity and total phenolic content. An evident color change, toward darker shades, did not affect the sensory scores among panelists. On the contrary, sensory properties scores, such as aroma and color, increased with the fortification degree increment [34].

### 4.4. Meta-Analysis of TDF, TPC, and Color Characteristics of Fortified Products

**Figure 1:**
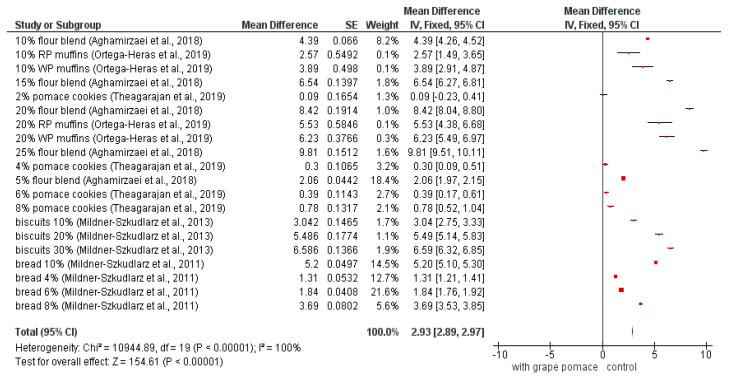
Meta-analysis of TDF in fortified products [20,23,43,45,75]; RP—red grape pomace; WP—white grape pomace; SE: standard error.

The results for TDF (total dietary fiber) content analyzed in different studies that included the product fortification by grape pomace are summarized in Figure 1. The meta-analysis of the total dietary fibers showed significant differences (*p* < 0.05) in all studies, except 2% and 4% pomace cookies [20]. The diamond does not touch the line of zero effect that indicates the overall significant difference. It means that the addition of grape pomace to these kinds of foodstuffs had no negligible impact on their TDF increase.

The content of TDF in grape pomace can go up to 88.7% [11], indicating that grape pomace could be successfully used as the source of TDF for the food product fortification.

**Figure 2:**
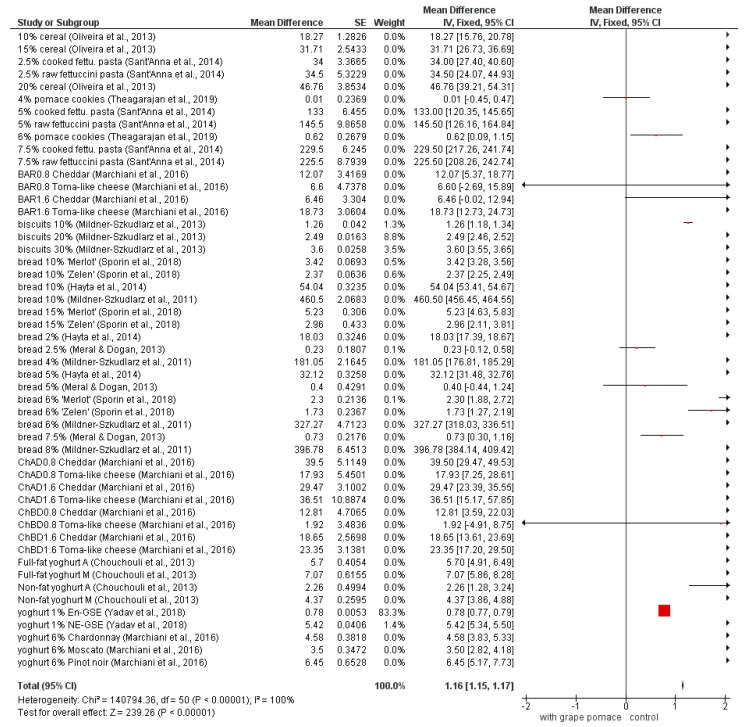
Meta-analysis of TPC in fortified products [20,21,23,45,46,47,50,53,69,71,73]; BAR—Barbera; ChAD—Chardonnay after distillation; ChBD—Chardonnay before distillation; En-GSE—encapsulated grape seed extract; NE-GSE—nonencapsulated grape seed extract; A—Agiorgitiko seed extracts; M—Moschofilero seed extracts.

Meta-analysis of total polyphenol content is shown in Figure 2. In summary, polyphenol content increased with the addition of grape byproducts. In a smaller number of fortified samples, statistical differences were not found in following studies: 4% pomace cookies [20], BAR0.8 Toma-like cheese, BAR1.6 Cheddar, CHBD0.8 Toma-like cheese [74], bread 2.5% and bread 5% [47]. All other fortified samples were significantly different (*p* < 0.05) from the samples without the addition of grape byproducts that served as control samples in studies. The diamond also showed the statistical difference and confirmed that the grape byproducts increased the TPC.

Since the approximately 70% of grape polyphenols remains in the grape pomace (accumulated in the process of winemaking) [12], the positive impact could be observed in grape pomace fortified products, it should be emphasized that grape pomace represents an important source of polyphenolic compounds.

**Figure 3:**
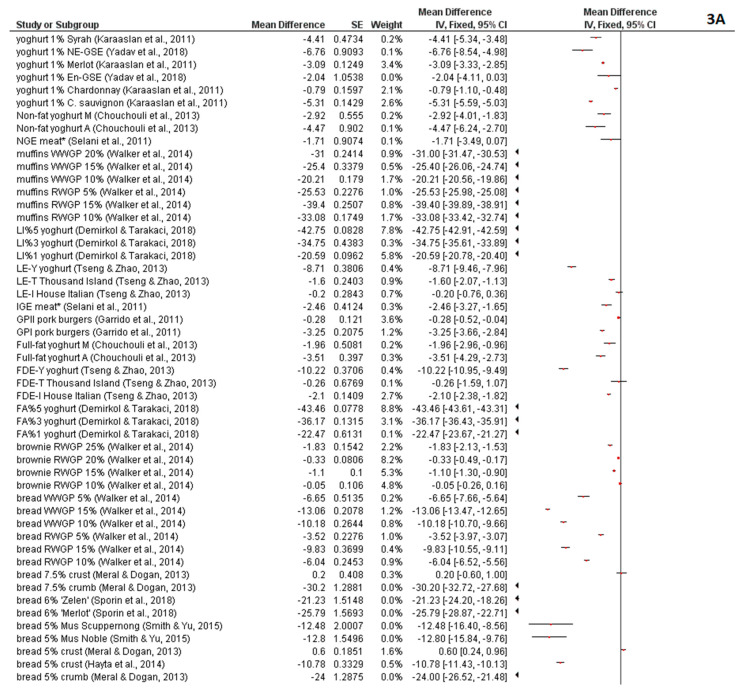

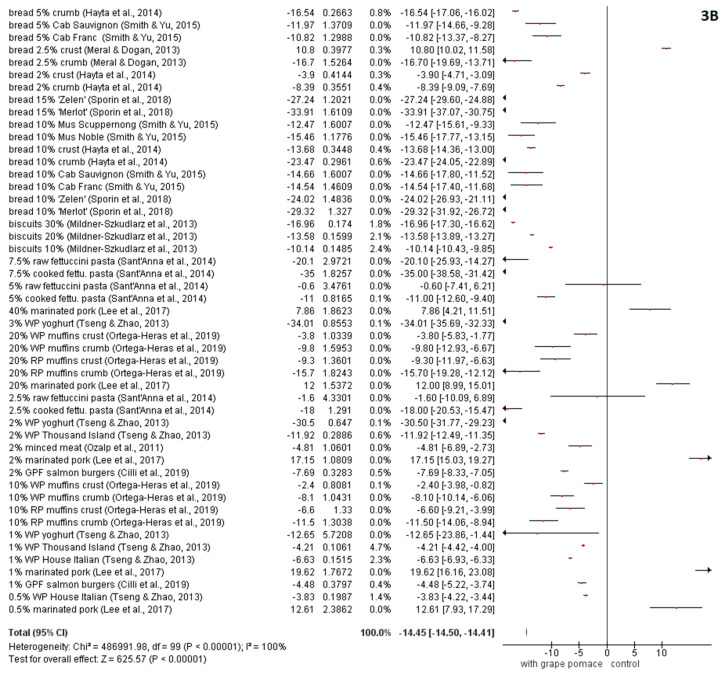
(**A**,**B**) Meta-analysis of *L* value in fortified products [13,19,21,22,23,43,44,46,47,48,53,60,63,64,69,70,72,73]; * En-GSE—encapsulated grape seed extract in yoghurt; NE-GSE—nonencapsulated grape seed extract in yoghurt; RP—red grape pomace; WP—white grape pomace; GPF—grape pomace flour; RWG—Pinot Noir wine grape pomace; WWGP—Pinot Grigio wine grape pomace; FA—forced air-dried grape pomace; LI—lyophilized grape pomace; A—Agiorgitiko seed extracts; M—Moschofilero seed extracts; LE—liquid pomace extract; FDE—freeze-dried pomace extract; GPI—grape pomace extract Type I (high-low instantaneous pressure (HLIP) + methanolic extraction); GPII—Grape pomace extract Type II (methanolic extraction; GPII); IGE—Isabel grape seed and peel extract; NGE—Niagara grape seed and peel extract.

**Figure 4:**
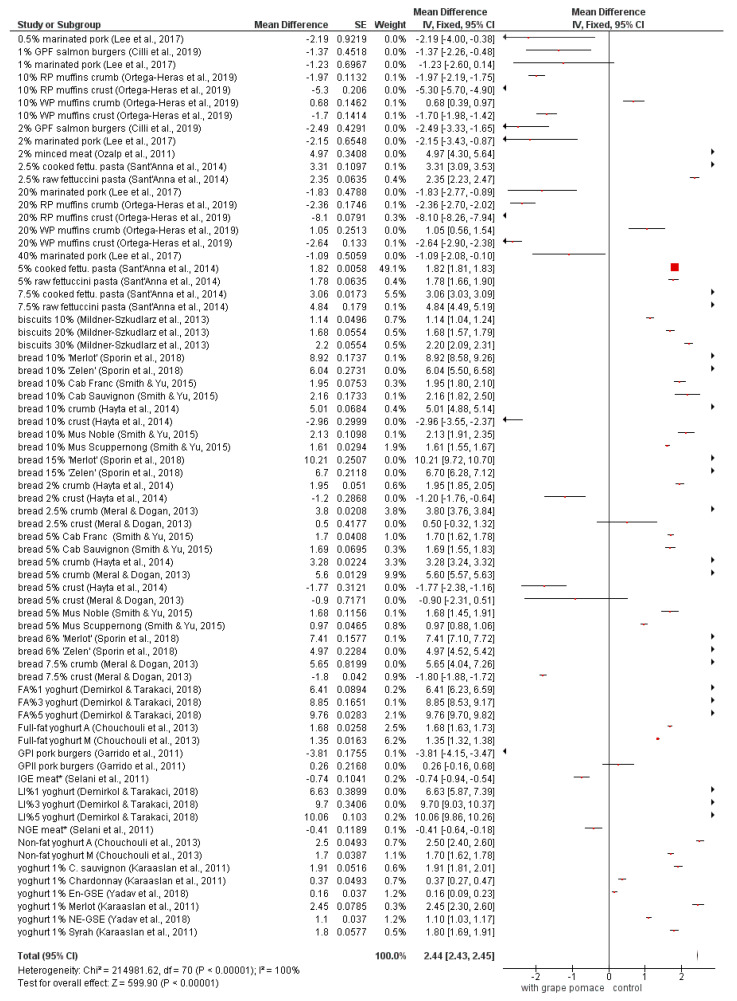
Meta-analysis of *a* value in fortified products [13,19,21,23,43,46,47,53,60,63,64,69,70,72,73]; * En-GSE—encapsulated grape seed extract in yoghurt; NE-GSE—nonencapsulated grape seed extract in yoghurt; RP—red grape pomace; WP—white grape pomace; GPF—grape pomace flour; FA—forced air-dried grape pomace; LI—lyophilized grape pomace; A—Agiorgitiko seed extracts; M—Moschofilero seed extracts; GPI—grape pomace extract Type I (high-low instantaneous pressure (HLIP) + methanolic extraction); GPII—Grape pomace extract Type II (methanolic extraction; GPII); IGE—Isabel grape seed and peel extract; NGE—Niagara grape seed and peel extract.

**Figure 5:**
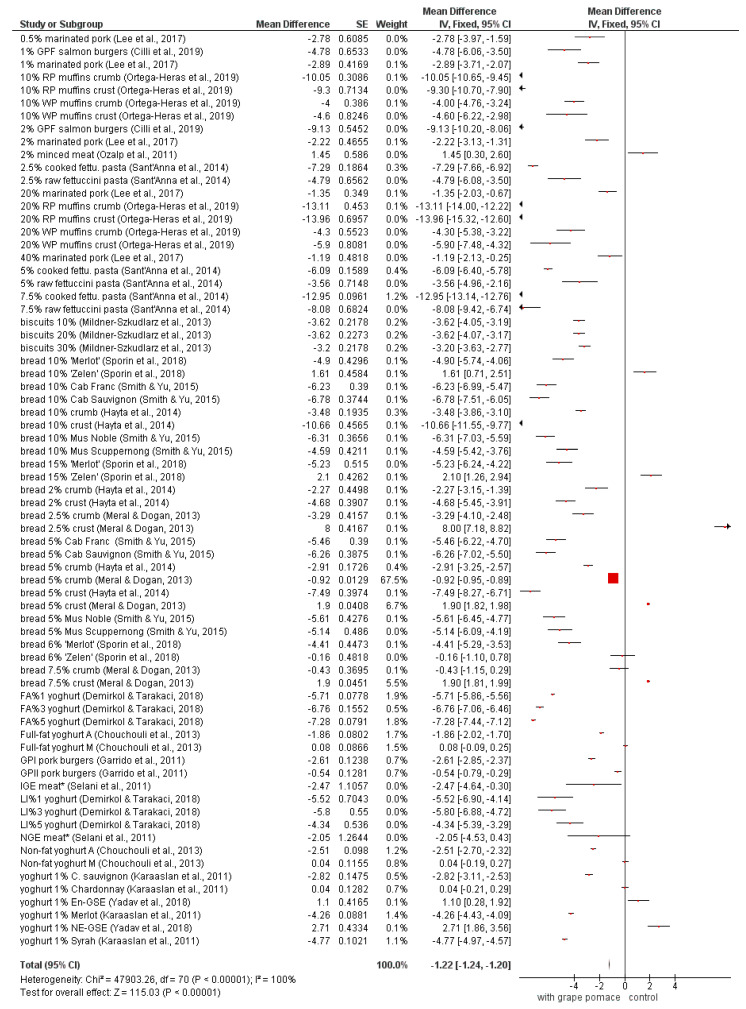
Meta-analysis of *b* value in fortified products [13,19,21,23,43,46,47,48,53,60,63,64,69,70,72,73]; * En-GSE—encapsulated grape seed extract in yoghurt; NE-GSE—nonencapsulated grape seed extract in yoghurt; RP—red grape pomace; WP—white grape pomace; GPF—grape pomace flour; FA—forced air dried grape pomace; LI—lyophilized grape pomace; A—Agiorgitiko seed extracts; M—Moschofilero seed extracts; GPI—grape pomace extract Type I (high-low instantaneous pressure (HLIP) + methanolic extraction); GPII—Grape pomace extract Type II (methanolic extraction; GPII); IGE—Isabel grape seed and peel extract; NGE—Niagara grape seed and peel extract.

The results of color characteristics (*L*, *a*, *b*) meta-analysis are summarized in Figure 3A,B, Figure 4, and Figure 5. Most of the experimentally-produced samples exhibited significantly lower *L* values (*p* < 0.05) (Figure 3A,B). According to the position of the diamond in the figures, which shows the average of all studies, the samples with the addition of grape pomace (generally) are darker than the control samples without grape pomace addition. Polyphenolics present in grape pomace are the main causative reason for the darker color of fortified samples. In red grape pomaces, the main representatives are tannins and anthocyanins. Hydroxycinnamic acids are main polyphenolic compounds in white grape pomace and they take part in oxidative browning reactions, contributing to the darker color formation [56,57]. The opposite effect for the change in *L* color value had all pork meat samples in the work of Lee et al. [60]. All treatments of those samples exhibited significantly higher (*p* < 0.05) *L* color values in comparison to control samples. The reason might be that the grape pomace was not directly incorporated into the samples, but they were marinated in different grape pomace solutions.

The color value *a* (Figure 4) shows different effects of the grape pomace addition. The diamond shows that the average of all studies was significantly (*p* < 0.05) higher than controls. The red color in red grape pomaces originates from anthocyanins, while in white grape pomaces it is connected with oxidative browning [56,57,60]. The negative *a* value (more green color) might be due to some other reactions of polyphenols and carotenoids [60].

The *b* color value is presented in Figure 5. Most of the studies reported a significant change in the decrease of *b* color value, confirmed by the diamond position, too. This means that samples become bluer and it was mainly caused by anthocyanins delphinidins [34]. The color in case of *b* value was influenced by the concentration degree, the type of fortified food, the preparation way and grape pomace type that was used. These findings are supported by the study conducted by Sporin et al. [21]. In their study bread was fortified by 10% incorporation of red (Merlot) and white (Zelen) grape pomace. The red grape pomace exhibited color that was significantly bluer (*p* < 0.05), while the white grape pomace significantly affected the change of color toward yellowish hue (*p* < 0.05). As mentioned before, flavonols present in white grape pomaces are responsible for a yellow color [56].

## 5. Conclusions

Grape pomace as a byproduct, accumulated mainly during wine production, represents a valuable source of important nutrients. The present systematic review showed the successful incorporation of grape pomace in different kinds of food commodities: plant food products, meat and fish products, and dairy products. The two most important ingredients, dietary fiber and polyphenols, were identified in the systematic review as the main bioactive compounds in grape pomace that can be used as fortification elements. The addition of grape pomace resulted in increased levels of total polyphenolic contents in all fortified final products, but these fortifications also lead to color changes (darker, reddish, and bluish). The increase of total polyphenolic contents also significantly increased the oxidative stability (especially meat and fish products) of fortified products and prolongated the shelf life. It should be stressed that a higher fortification degree, which included a higher grape pomace concentration in different products, mainly adversely affected textural and sensory characteristics. The findings confirmed in the systematic review indicate the undeniable positive impact of grape pomace on all types of food commodities. Certainly, sensory properties can be affected differently and individual food commodities have to be tested separately for possible grape pomace fortification.

## Figures and Tables

**Table 1:** Proximate composition of grape pomace based on dry weight (data from Bender et al. [9]; Mohamed Ahmed et al. [18]; Cilli et al. [19]; Beres et al. [12]; Theagarajan et al. [20]; Šporin et al. [21]; Tseng et al. [22]; Mildner-Szkudlarz et al. [23]; Acun and Gul [11]; Nagarajaiah et al. [24]; Deng et al. [25]; Llobera et al. [26]; Winkler et al. [27]; Sousa et al. [2]; Anđelković et al. [28]; Rondeau et al. [29]; Jin et al. [17]; Javier et al. [30]).

Compounds	Quantity g/100 g	Compounds	Quantity mg/100 g
Ash	1.73–9.10	Na	87–244
Protein	3.57–14.17	K	1184–2718
Fat	1.14–13.90	Mg	92–644
Total dietary fiber	17.28–88.70	Ca	91–961
Insoluble fiber	16.44–63.70	Mn	6–1356
Soluble fiber	0.72–12.78	Fe	5–5468
Carbohydrates	12.20–40.53	Zn	2–2254
TPC *	0.28–8.70	Cu	39–130
Fructose	0.38–8.91	P	4–3157
Glucose	0.21–26.34		

* Total polyphenolic content.

**Table 2:** Effect of addition of grape pomace to the plant origin products.

Product	Conditions	Major Findings
Muffins, Bender et al. [4]	Replacement of wheat flour with 5, 7.5 and 10% of grape skin flour	↑ Increased dietary fiber content and well accepted among the consumers ↓ Change in color and textural properties
Muffins, Ortega-Heras et al. [43]	Replacement of whole wheat flour with 10 and 20% of grape pomace	↑ Increase dietary fiber content and good sensory acceptability ↓ Change in color and textural properties
Cookies, Theagarajan et al. [20]	Replacement of wheat flour with 2, 4, 6 and 8% of grape pomace	↑ Increased polyphenolic content and lipid oxidation and textural stability during storage time ↓ Significant change in sensory properties
Bread, Sporin et al. [21]	Addition of 6, 10 and 15% of grape pomace based on the wheat flour content	↑ Increased polyphenolic content and antioxidant activity ↓ Darker color
Bread, Walker et al. [44]	Replacement of wheat flour with 5 10 and 15% of grape pomace	↑ Increased total phenolic content, radical scavenging activity and total dietary fiber ↓ Change in color, texture
Muffins, Walker et al. [44]	Replacement of up to 20% of flour with grape pomace	↑ Increased total phenolic content, radical scavenging activity and total dietary fiber ↓ Change in color, texture
Brownie, Walker et al. [44]	Replacement of up to 25% of flour with grape pomace	↑ Increased total phenolic content, radical scavenging activity and total dietary fiber ↓ Change in color, texture
Sourdough rye bread, Mildner-Szkudlarz et al. [45]	Addition of 4, 6, 8 and 10% of grape pomace to the bread mixture	↑ Increased dietary fiber, total phenolic content and antioxidant activity ↓ Textural changes in the final product
Biscuits, Mildner-Szkudlarz et al. [23]	Replacement of wheat flour with 10, 20 and 30% of grape pomace	↑ Increased dietary fiber, polyphenolic content and antioxidant activity ↓ Decreased hardness and change in color
Bread, Hayta et al. [46]	Replacement of flour with 2, 5 and 10% of grape pomace	↑ Increased total phenolic content and anti-radical activity ↓ Increased hardness and darkness of the product
Bread, Meral and Dogan, [47]	Replacement of wheat flour with 2.5%, 5% and 7.5% of grape seed flour	↑ Increased antioxidant activity and phenolic content. Improved rheological properties ↓ Change in color
Bread, Smith and Yu [48]	replacement of 5% and 10% of wheat flour with grape pomace	↑ Increased antioxidant activity, total phenolic content and dietary fiber content. ↓ Reduced loaf volume, darker color, and harder texture
Biscuits, Aksoylu et al. [49]	Incorporation of 5% of grape seed powder into biscuit recipe	↑ Increase of total phenolic content and antioxidant activity ↓ Darker color
Extruded cereals, Oliveira et al. [50]	Replacement of 10%, 15% and 20% of corn grits with grape skin and seed powder	↑ Increase of total phenolic content and crude fibers ↓ Decrease in hardness
Cereal bars, noodles, pancakes, Rosales Soto et al. [51]	Incorporation of 5% to 30% of grape seed flour in product recipe	↑ Increase of antioxidant activity and polyphenolic content
Pasta, Marinelli et al. [52]	Preparation of pasta using the grape pomace water extract	↑ Increase of antioxidant activity and total phenolic content
Pasta, Sant’Anna et al. [53]	Addition of 2.5, 5 and 7.5% of grape pomace powder in fettuccine pasta preparation	↑ Increase of antioxidant activity and total phenolic content ↓ Change in color
Tomato puree, Lavelli et al. [54]	Addition of 3.2% of grape skin powder to the tomato puree	↑ Increase of antioxidant activity and total phenolic content
Tea infusions, Bekhit et al. [15]	Preparation of tea infusion out of grape skins	↑ Refreshing sensory perception ↓ Weaker antioxidant activity in comparison to the other tea mixtures
Rice, Balbinoti et al. [5]	Addition of grape pomace flour in the process of parboiling of the rice, GP rice ratio 1:2	↑ Improving the antioxidant activity ↓ Change in color
Pasta, Gaita et al. [55]	Replacement of wheat flour with 3, 6 and 9% of grape skins flour	↑ Increase of antioxidant activity total phenolic content; better sensory evaluation

↑ positive effect ↓ negative effect.

**Table 3:** Effect of addition of grape pomace to the meat and fish products.

Product	Conditions	Major Findings
Salmon burger, Cilli et al. [19]	Addition of 1 and 2% of grape pomace flour to the burger recipe	↑ Increased dietary fiber content and storage stability ↓ Decrease in sensory properties
Pork loin marinade, Lee et al. [60]	Soaking of pork loin in 0.5, 1, 2, 20 and 40% grape pomace solution	↑ Inhibits the lipid oxidation and microorganisms growth
Pork burger, Garrido et al. [13]	Addition of 0.06% of grape pomace extract to the product weight	↑ Inhibition of lipid oxidation and enhanced color stability
Frankfurters, Özvural and Vural [61]	Addition of up to 0.5% of grape seed extract to the recipe	↑ Decreased lipid oxidation ↓ Change in sensory and textural properties
Pork sausages, Ryu et al. [62]	Incorporation of 0.5 and 1% of grape pomace into the recipe	↑ Decreased lipid oxidation ↓ Change in color
Chicken meat, Selani et al. [63]	Addition of grape pomace extract to achieve TPC 60 mg/kg in meat	↑ Decreased lipid oxidation in raw and cooked meat. ↓ Change in color and flavor
Minced fish, Ozalp et al. [64]	Addition of 2% of grape seed extract to the minced fish muscle	↑ Decreased lipid oxidation ↓ Change in color
Chicken meat, Shirahigue et al. [65]	Addition of grape pomace extract to achieve 10, 20, 40 and 60 mg/kg TPC in meat	↑ Decreased lipid oxidation
Chicken meat, Rababah et al. [66]	Soaking of chicken breasts in 0.25% grape pomace extract	↑ Decreased lipid oxidation, improved texture properties
Minced fish muscle, Sánchez-Alonso et al. [67]	Addition of 2 and 4% of grape pomace to the minced fish muscle	↑ Decreased lipid oxidation during storage, increased antioxidant activity

↑ positive effect ↓ negative effect.

**Table 4:** Effect of addition of grape pomace to the dairy products.

Product	Conditions	Major Findings
Fermented milk, Frumento et al. [68]	Addition of 20 g/L of grape pomace to the milk base	↑ Increase of antioxidant activity and phenolic content, and accelerated fermentation
Yogurt, Chouchouli et al. [69]	Addition of 100mg of dry seed extract in 150 mL of milk	↑ Increase of antioxidant activity and total phenolic content ↓ Change in color
Yogurt, Karaaslan et al. [70]	Addition of 1% of grape pomace extract to yogurt formulation	↑ Increase of antioxidant activity and total phenolic content
Yogurt, Marchiani et al. [71]	Addition of 6% of grape skin flour to yogurt formulation	↑ Increase of antioxidant activity and total phenolic content ↓ Decreased liking among the consumers
Yogurt, Demirkol et al. [72]	Addition of 1, 3 and 5% of grape pomace to the milk before the fermentation	↑ Increased polyphenolic content and antioxidant activity. Sensory acceptable products. ↓ Decreased viscosity
Yogurt, Yadav et al. [73]	Addition of 1% of grape seed extract to the milk before the fermentation	↑ Increased total phenolic content and antioxidant capacity ↓ Change in color
Yogurt and salad dressing, Tseng et al. [22]	Addition of 1, 2 and 3% of grape pomace to the yogurt product	↑ Increased polyphenolic content and antioxidant activity ↓ Darker product
Cheese, Marchiani et al. [74]	Incorporation of 0.8 and 1.6% of grape pomace into cheese formulation	↑ Increase of antioxidant activity and total phenolic content
Ice cream, Vital et al. [34]	Addition of 2.5, 5 and 10% of grape pomace to the ice cream formulation	↑ Increase of antioxidant activity total phenolic content

↑ positive effect ↓ negative effect.
